# Red cell distribution width as a prognostic marker for complications of community-acquired pneumonia in children: a comparison with Proadrenomedullin and Copeptin

**DOI:** 10.1186/s12890-023-02686-z

**Published:** 2023-10-04

**Authors:** Asmaa N. Moustafa, Hend M. Moness, Marwa Waly Eldin Ali

**Affiliations:** 1 Department of Pediatrics, Faculty of Medicine, Minya University Hospital, Minya, 61111 Egypt; 2 Department of Clinical Pathology, Faculty of Medicine, Minya University, Minya, Egypt

**Keywords:** Community-acquired pneumonia, Pro adrenomedullin, Copeptin, Red cell distribution width

## Abstract

**Background:**

Community-acquired pneumonia (CAP) is the most common leading cause of morbidity and mortality in children; so, early identification of patients with CAP, who are at risk of complications or high mortality, is very critical to identify patients who need early admission to the intensive care unit.

**Purpose of the study:**

To explore the prognostic value of Red Cell Distribution Width (RDW), Proadrenomedullin and Copeptin in the prediction of complicated CAP in children.

**Methods:**

99 children were enrolled in the study, which was done at the Pediatric Department of Minia University Hospital. Measurement of serum Proadrenomedullin, Copeptin, and RDW was done to all participating children in the first 24 h of admission. Assessment of the severity of CAP was done using the Pediatric Respiratory Severity Score (PRESS).

**Results:**

The values of RDW, Proadrenomedullin, and Copeptin were significantly higher in the complicated CAP group than in the uncomplicated one (P value < 0.01). There were significant positive correlations between RDW and Proadrenomedullin with PRESS (r 0.56 for both). For the prediction of complications, RDW at cutoff point > 17.4, has 77.7% of sensitivity and 98.6% of specificity, followed by Pro ADM at cutoff point > 5.1 nmol/L, of 74% of sensitivity and 90.2% of specificity. For the prediction of mortality, RDW at cutoff point > 17.4 has 81.25% of sensitivity and 89.16% of specificity.

**Conclusion:**

The RDW is a reliable predictor of poor outcomes in pediatric CAP.

## Introduction

Community-acquired pneumonia (CAP) is a leading cause of childhood morbidity and mortality all over the world [1]. Killing about 740,180 children younger than 5 years in 2019, responsible for 14% of all deaths of children under 5 years old and 22% of all deaths in children aged 1–5 years, and most of these deaths occur in developing countries [2]. Also, nearly 10% of child deaths under the age of 5 in Egypt are caused by pneumonia and other acute respiratory infections [3].

Recently, the prognostic usefulness of various biomarkers in children with CAP has been evaluated. These biomarkers are pro-adrenomedullin (pro-ADM) and Copeptin (Copeptin), which are peptides co-synthesized together in endothelial cells and pituitary gland respectively [4]. These peptides have powerful vasodilation and bactericidal effects, immune-modulating and metabolic properties [5, 6].

Red cell distribution width (RDW) is a parameter for measuring variability in red blood cell sizes. Many researchers have studied RDW as a prognostic marker in various diseases such as septic shock, acute kidney injury, pulmonary hypertension, pulmonary embolism and community-acquired pneumonia [7]. RDW is routinely included in the automated complete blood count (CBC) analyzer and has no additional costs; consequently, evaluating RDW as a prognostic marker for CAP is beneficial, especially for developing countries such as Egypt. The objective of this study is to evaluate the prognostic value of RDW, proadrenomedullin and Copeptin in the prediction of complications of CAP in children.

## Patients and methods

### Study design and setting

This prospective hospital-based study was carried out at Minia University Children’s Hospital over the period from 1st September 2020 to 31st January 2021.

The study was explained in detail to the parents or legal guardians of the participating children, and written consent forms were taken from them.

### Selection of patients

The study included 99 children who were hospitalized with CAP in the Respiratory In-patient Unit or the Pediatric Intensive Care Unit (PICU) of Minia University Children’s Hospital. Eligible patients were between 1 month and 120 months old. Patients were diagnosed with CAP, according to WHO diagnostic criteria including; (1) increased respiratory rate (rate > 60 breaths/ minute if aged less than 2 months, > 50 breaths/minute if aged 2–11 months, and > 40 breaths/minute if aged 12– 59 months); (2) lower chest wall indrawing (severe pneumonia); or (3) cyanosis and/or inability to eat or drink (very severe pneumonia) [8]. Chest X-rays were analyzed independently by two radiologists, according to the WHO criteria [9].

We excluded children with an underlying chronic respiratory disease (bronchial ectasia, bronchopulmonary dysplasia, cystic fibrosis), children who were assumed to have aspiration pneumonia from preceding aspiration events, and those with a chronic neurologic disease or a known immunodeficiency syndrome. Evaluation of the severity of CAP by using the Pediatric Respiratory Severity Score (PRESS). The PRESS assessed tachypnea, wheezing, retraction (accessory muscle use), SpO_2_, and feeding difficulties, with each component given a score of 0 or 1, and total scores were classified as mild (0–1), moderate (2–3), or severe (4–5) [10] for each child at admission to the ER department by the attending physician. All patients admitted in the ER department then cases who fulfilled the criteria for Pediatric Intensive Care Unite admission admitted to PICU while the remaining patients referred to pulmonology unit. A chest X-ray was done for every child with CAP.

The followings were the indications for admission to Pediatric Intensive Care Unit (PICU): Failure to maintain oxygen saturations > 92% with FiO2 60%, clinical features of shock, increasing respiratory and heart rates with severe respiratory distress and exhaustion, with or without raised pCO2 and recurrent apnoea or slow irregular breathing.

In the present study, CAP complications were considered if any of the following was present at the time of admission: pleural effusion, empyema, lung abscess, pneumothorax, respiratory failure or signs of severe sepsis or septic shock according to the International Pediatric Sepsis Consensus Conference criteria in 2005 [11].

### Data collection

Medical history was obtained through interviews with the parents or legal guardians. Age; sex; comorbidities (including heart failure, hepatic, renal, and central nervous system diseases); vital signs (blood pressure, heart rate, respiratory rate, and body temperature). Laboratory data: CBC including RDW, serum pro-adrenomedullin and serum Copeptin were done to all participating children in the first 24 h of admission.

### Blood sampling

Under aseptic conditions, five ml of venous blood was withdrawn from all patient groups during the first 24 h after ER department admission and before starting antibiotic therapy.

Two ml of blood was put in an EDTA tube for the determination of CBC. After that, the EDTA tube was centrifuged and expressed plasma was kept on-70 °C for later assessment of proadrenomedullin. The remaining three ml was put in a plain tube and left to clot and then centrifuged. The expressed serum was frozen at -70 °C for Copeptin assay later.

## Methods

- CBC (including RDW) was performed using an automated blood counter (Sysmex KX-21 N).
- The plasma Proadrenomedullin level was assayed by an enzyme-linked immunosorbent assay kit (using DRG International Inc., USA kit. The lower detection level is 0.33nmol/L.
- Lastly, serum Copeptin (vasopressin –neurophysin2) level (kits were supplied from EIAab- China Science Co.Ltd) www.eiaab.com”. The detection range was 15- 1000pg/ml.

### Statistics

Data were analyzed using the Statistical Package for the Social Sciences (SPSS), Version 21 for Windows. Descriptive statistics were expressed as mean ± standard deviation (range), while categorical data were presented as frequency and percentage (%). Comparisons for continuous, normally distributed variables were performed via a T-test or Mann–Whitney test for the not normally distributed data. However, comparisons of categorical data were performed via a Chi-square test. Correlations between biomarkers and PRESS were assessed via a Pearson’s and Spearman’s rank correlation test. Simple logistic regression and multiple stepwise logistic regression analyses were used to evaluate the relation of biomarkers and complications of CAP and also the mortality. The predictive value of biomarkers for complications of CAP and the mortality was assessed by receiver operator characteristic (ROC) with an area under the curve (AUC). The probability (P) value < 0.05 was considered significant and P. Value of < 0.01 was considered as highly significant.

## Results

This prospective hospital-based study, including 99 children suffering from CAP admitted to the PICU of Minia University Children’s Hospital over the period between 1st September 2020 to 31st January 2021. Among those 99 patients, about 27 children (27.3%) showed complicated CAP (8 children went through septic shocks (8.3%), 1 child went through pneumothorax (1%), 12 children went through respiratory failures (12.1%), 2 children went through lung abscess (2%) and 4 children went through effusion (4%)). The 30-day mortality rate was 16.2% (16), 25 patients admitted to PICU (27.3%) as shown in Table 1.

**Table 1:** Basal and laboratory characteristics of the studied group:

		Descriptive statistics N = 99
Age (months)	*Median (IQR)*	4(2–18)
Sex	*Male* *Female*	60(60.6%) 39(39.4%)
Weight (Kg)	*Median (IQR)*	6(5–9)
Length of hospital stay (days)	*Median (IQR)*	7(5–10)
Cyanosis	*-Ve* *+Ve*	91(91.9%) 8(8.1%)
Complications	*-Ve* *+Ve*	72(72.7%) 27(27.3%)
Complications	*No* *Septic shock* *Respiratory failure* *Pneumothorax* *Lung abscess* *Effusion*	72(72.7%) 8(8.1%) 12(12.1%) 1(1%) 2(2%) 4(4%)
PRESS	*Mean ± SD*	3.3 ± 0.9
pro-adrenomedullin nmol/l (pro-ADM)	*median (IQR)*	4.2(2.4–5.2)
Copeptin (Copep) pmol/l	*median (IQR)*	640(450–860)
Red Cell Distribution Width (RDW)	*Mean ± SD*	15.9 ± 1.5
TLC	*median (IQR)*	10(6.3–15.5)
Outcome	*Improved* *Died* **(30 days in-Hospital mortality)**	83(83.8%) 16(16.2%)
PICU Admission	*-Ve* *+Ve*	74(74.7%) 25(25.3%)

IQR: interquartile range, PRESS : pediatric respiratory severity score, TLC: Total leukocytic count, PICU pediatric intensive care unit

The Comparison between Group (I) and Group (II) (uncomplicated versus complicated CAP) regarding baseline characteristics (details in Table 2) showed that males assume a high percentage in the uncomplicated group (**56.9**%) with a greater percentage in the complicated group (70.4%). The length of hospital stay was longer in the complicated group than the uncomplicated group (Median& IQR 11(9–16) vs. 5.5( 4–8) respectively).

**Table 2:** Comparison between complicated and uncomplicated community acquired pneumonia (CAP) groups as regard baseline characteristics

		Group (I) Uncomplicated CAP(n = 72)	Group (II) Complicated CAP (N = 27)	P value
Age (months)	*Median (IQR)*	4(2–12)	5(2–48)	*0.218*
Sex	*Male* *Female*	41(56.9%) 31(43.1%)	19(70.4%) 8(29.6%)	*0.223*
Weight (Kg)	*Median(IQR)*	6(5–8)	5(4.5–18)	*0.893*
Length of Hospital Stay. (days)	*Median(IQR)*	5.5(4–8)	11(9–16)	< 0.001*
Cyanosis	*-Ve* *+Ve*	71(98.6%) 1(1.4%)	20(74.1%) 7(25.9%)	< 0.001*

Mann Whitney test for non-parametric quantitative data between the two groups

Chi square test or Fisher’s exact test for qualitative data between the two groups

*: Significant level at P value < 0.05

As shown in Table 3, the comparison between the complicated CAP group and uncomplicated CAP group regarding studied markers shows that there were significant differences in the values of RDW, Pro ADM and Copeptin between the two groups.

**Table 3:** Comparison between complicated CAP and uncomplicated CAP as regard the studied markers

Variable		Group (I) Uncomplicated CAP(n = 72)	Group (II) Complicated CAP (N = 27)	P value
PRESS	*Mean ± SD*	3 ± 0.9	4 ± 0.8	< 0.001*
pro-adrenomedullin nmol/l (pro-ADM)	*Median (IQR)*	3.5(1.9–4.3)	5.3(5.1–5.8)	< 0.001*
Copeptin (Copep) pmol/l	*Median (IQR)*	550(442.5–750)	870(550–1120)	< 0.001*
Red Cell Distribution Width (RDW)	*Mean ± SD*	15.2 ± 0.9	17.8 ± 0.9	< 0.001*
TLC	*Median (IQR)*	9(5.7–13)	16(15.2–22)	< 0.001*

Mann Whitney test for non-parametric quantitative data between the two groups

Independent samples T test for parametric quantitative data between the two groups

Fisher’s exact test for qualitative data between the two groups

*: Significant level at P value < 0.05

PRESS: pediatric respiratory severity score

The mean values in the complicated versus uncomplicated groups are as follows (RDW (Mean ***±*** SD): 17.8 ***±*** 0.9 vs. 15.2 ***±*** 0.9, Pro ADM(Median IQR) : 5.3 (5.1–5.3) vs. 3.5(1.9–4.3) and Copeptin(Median IQR) 870(550–1120) vs. 550 (442.5–750) respectively).

The studied biomarkers in addition to other variables were further analyzed via a simple logistic regression analysis for prediction of complications, and it was found that Pro-ADM was the most significant one (OR 7.83, p **< 0.001**), followed by RDW (OR 7.53, p **< 0.001**), the length of hospital stay (OR 1.5, p **< 0.001)**, Copeptin (OR 1.003, p **=** 0.001), and lastly Total leukocytic count TLC (OR 1, p **< 0.001)** as is shown in Table 4.

**Table 4:** Simple logistic regression analysis for prediction of complications

	OR	95% CI	P value
Duration of Hospital Stay (days)	1.52	1.28–1.8	< 0.001*
Cyanosis			
-Ve	Ref.		
+Ve	24.85	2.89-214.03	0.003*
PRESS	4.46	2.19–9.08	< 0.001*
Pro ADM	7.83	3.03–20.28	< 0.001*
Copeptin	1.003	1.001–1.005	0.001*
RDW	7.53	3.39–16.75	< 0.001*
TLC	1	1–1	< 0.001*

OR: Odds Ratio

CI: Confidence Interval

Ref.: Reference

*: Significant level at P value < 0.05

Further analysis of the same variables via a multiple stepwise logistic regression analysis for prediction of complications was done, and it was found that RDW was the most significant variable (Adjusted odds ratio) AOR( 6.31 p **< 0.001**), followed by the length of hospital stay ( Adjusted odds ratio) AOR( 1.33 p-value 0.04) as is shown in Table 5.

**Table 5:** Multiple stepwise logistic regression analysis for prediction of complications

	AOR	95% CI	P value
Length of Hospital Stay (day)	1.33	1.04–1.69	0.021*
RDW	6.31	2.48–16.09	< 0.001*

AOR: Adjusted Odds Ratio

CI: Confidence Interval

*: Significant level at P value < 0.05

Associations between PRESS and studied biomarkers were done revealing a significant moderate association between Pro ADM and PRESS (r 0.56 and p **< 0.001**), a significant moderate association between RDW and PRESS (r 0.56 and p **< 0.001)** and a significant mild association between Copeptin and PRESS (r 0.44 and p < 0.01) as is shown in Table 6.

**Table 6:** Correlation between PRESS and studied biomarkers

	PRESS	
	r	P value
pro-adrenomedullin nmol/l (pro-ADM)	0.564	< 0.001*
Copeptin (Copep) pmol/l	0.445	< 0.001*
Red Cell Distribution Width (RDW)	0.564	< 0.001*
TLC	0.329	0.001*

Pearson’s Correlation

*: Significant level at P value < 0.05

TLC: Total leukocytic count

**Fig. 1:**
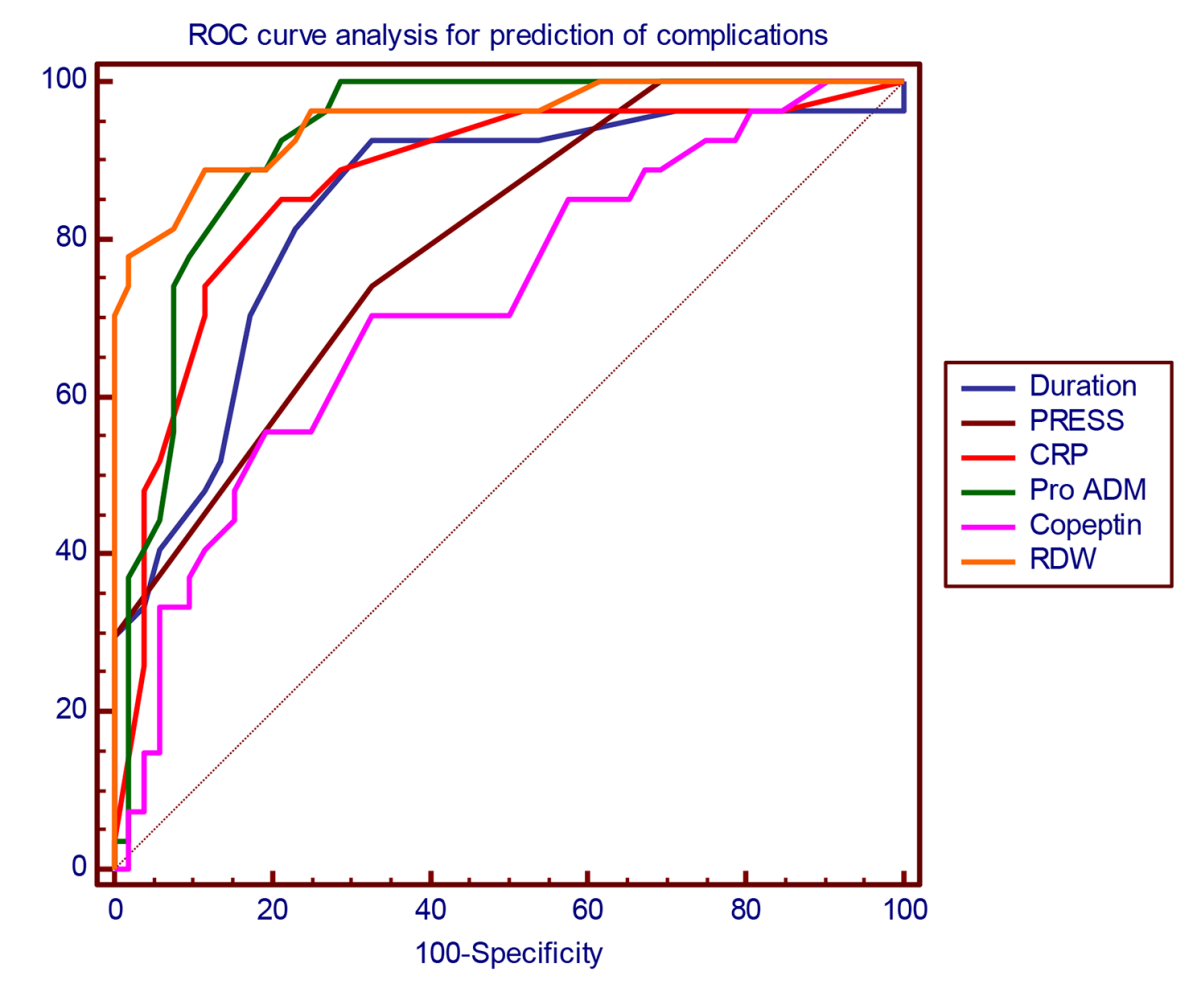
Roc curve for prediction of complications in community acquired pneumonia in children. This figure showing that RDW was the most accurate one for prediction of complications at the optimal cut-off point 17.4, with sensitivity of 77.7% and specificity of 98.6%, followed by Pro ADM at cut-off point 5.1 nmol/L with sensitivity of 74% and specificity of 90.2%, and lastly by Copeptin at cut-off point 780 pmol/l with sensitivity of 55.5% and specificity of 83.3%

ROC analysis to elucidate the usefulness of the studied markers for prediction of complications of CAP was done, and it revealed that RDW was the most accurate one for prediction of complications at the optimal cutoff point 17.4, with sensitivity of 77.7% and specificity of 98.6%, followed by Pro ADM at cutoff point 5.1 nmol/L with sensitivity of 74% and specificity of 90.2%, and lastly by Copeptin at cutoff point 780 pmol/l with sensitivity of 55.5% and specificity of 83.3%, as is shown in Table 7; Fig. 1.

**Table 7:** Receiver operating characteristic (ROC) curve analysis for prediction of complications

	Duration of Hospital Stay	PRESS	Pro ADM	Copeptin	RDW
Cutoff	> 9	> 3	> 5.1	> 780	> 17.4
AUC	0.863	0.786	0.909	0.730	0.959
95% CI	0.779–0.924	0.692–0.862	0.835–0.958	0.632–0.815	0.899–0.988
P value	< 0.001*	< 0.001*	< 0.001*	< 0.001*	< 0.001*
Sensitivity	70.37	74.07	74.07	55.56	77.78
Specificity	86.11	65.28	90.28	83.33	98.61
PPV	65.5	44.4	74.1	55.6	95.5
NPV	88.6	87	90.3	83.3	92.2
Accuracy	81.8	67.7	85.9	75.8	92.9

AUC: Area Under Curve

CI: Confidence Interval

PPV: Positive Predictive Value

NPV: Negative Predictive Value

*: Significant Level at P value < 0.05

The studied biomarkers, in addition to other variables, were further analyzed via a simple logistic regression analysis for prediction of mortality, and it was found that RDW was the most significant one (OR 5.96, p **< 0.001**), followed by Pro-ADM (OR 4.52, p **0.001**), length of hospital stay ( OR 1.24, p **0.001), PRESS (**OR 3, p = 0.003), TLC (OR 1, p 0.001), and lastly Copeptin (OR 1.002, p **=** 0.065), as is shown in Table 8.

**Table 8:** Simple logistic regression analysis for prediction of mortality

	OR	95% CI	P value
Weight	0.92	0.8–1.06	*0.254*
Duration of Hospital Stay	1.24	1.09–1.42	0.001*
PRESS	3	1.46–6.16	0.003*
Pro ADM	4.52	1.88–10.87	0.001*
Copept	1.002	1-1.003	*0.065*
RDW	5.96	2.34–15.17	< 0.001*
TLC	1	1–1	0.001*

OR: Odds Ratio

CI: Confidence Interval

Ref.: Reference

*: Significant leel atP value < 0.05

Further analysis of the same variables via a multiple stepwise logistic regression analysis for prediction of mortality was done, and it was found that RDW was the most significant variable (Adjusted odds ratio )AOR( 5.35 p < 0.001), as is shown in Table 9.

**Table 9:** Multiple stepwise logistic regression analysis for prediction of mortality

	AOR	95% CI	P value
RDW	5.35	2.09–13.67	< 0.001*

AOR: Adjusted Odds Ratio

CI: Confidence Interval

*: Significant level at P value < 0.05

**Fig. 2:**
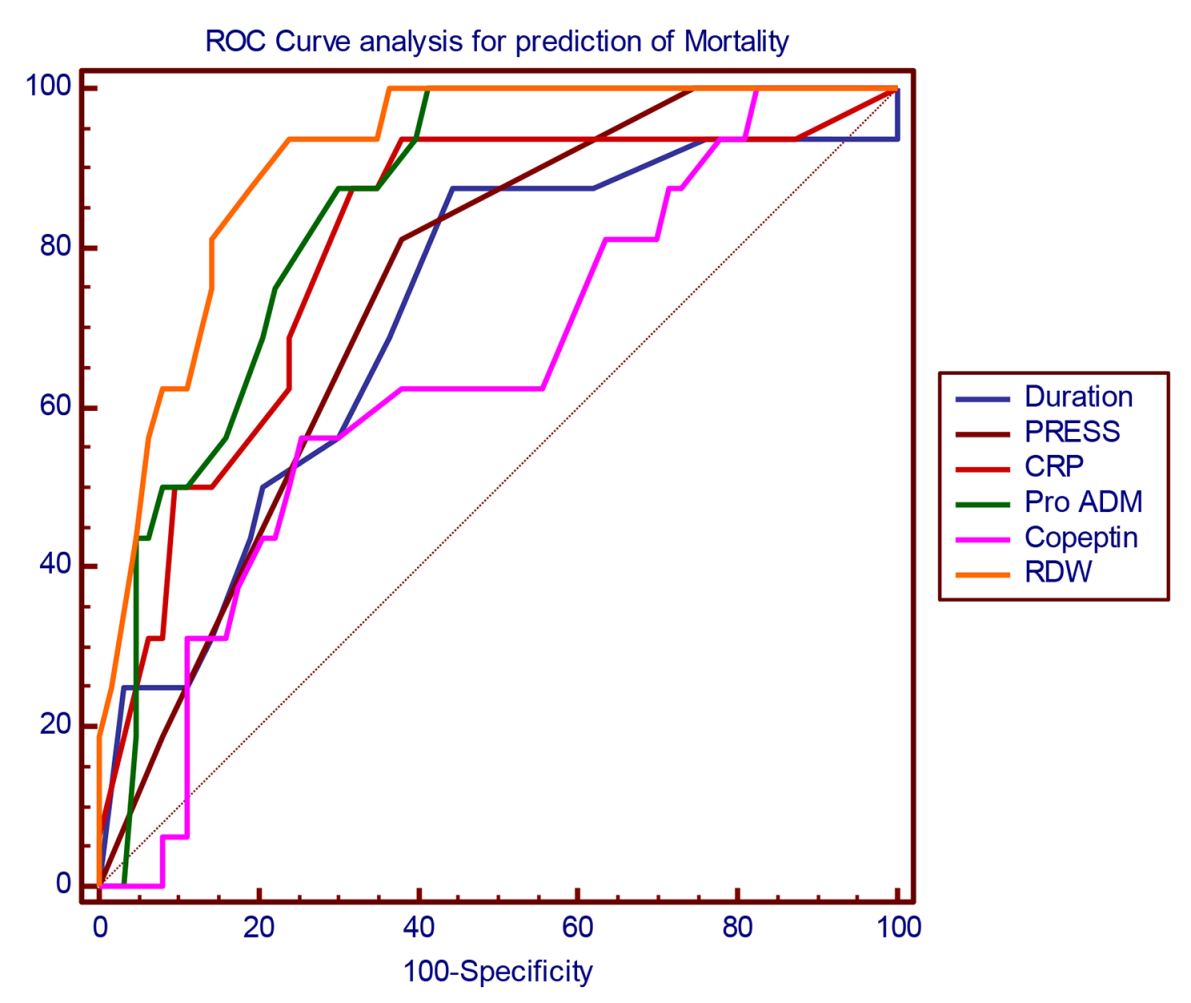
Roc curve for prediction of mortality in community acquired pneumonia in children. This figure revealing that RDW was the most accurate prognostic biomarker at cut-off point 17.4% with sensitivity of 81% and specificity of 89%, followed by Pro ADM at cut-off point 4.75 nmol/l with sensitivity of 87.5% and specificity of 68.6%, Copeptin at cut-off point 780 pmol/l with sensitivity of 56% and specificity of 78%, length of hospital stay (more than 7 days) at cut-off point with sensitivity of 87.5% and specificity of 62.6%, and lastly PRESS at cut-off point more than 3 with sensitivity of 81.2% and specificity of 61.4%

The other ROC model was done to determine the prognostic accuracy of the studied biomarkers for the prediction of mortality. It was found that RDW was the most accurate prognostic biomarker at cutoff point 17.4% with sensitivity of 81% and specificity of 89%, followed by Pro ADM at cutoff point 4.75 nmol/l with sensitivity of 87.5% and specificity of 68.6%, Copeptin at cutoff point 780 pmol/l with sensitivity of 56% and specificity of 78%, length of hospital stay (more than 7 days) at cutoff point with sensitivity of 87.5% and specificity of 62.6%, and lastly PRESS at cutoff point more than 3 with sensitivity of 81.2% and specificity of 61.4%, as is shown in Table 10; Fig. 2.

**Table 10:** Receiver operating characteristic (ROC) curve analysis for prediction of mortality

	Duration of hospital stay	PRESS	Pro ADM	Copeptin	RDW
*Cutoff*	> 7	> 3	> 4.7	> 780	> 17.4
AUC	0.752	0.747	0.852	0.666	0.931
95% CI	0.655–0.834	0.650–0.829	0.767–0.916	0.564–0.757	0.863–0.972
P value	< 0.001*	< 0.001*	< 0.001*	0.024*	< 0.001*
Sensitivity	87.5	81.25	87.5	56.25	81.25
Specificity	62.65	61.45	68.67	78.31	89.16
PPV	31.1	28.9	35	33.3	59.1
NPV	96.3	94.4	96.6	90.3	96.1
Accuracy	66.7	64.6	71.7	74.7	87.9

AUC: Area Under Curve

CI: Confidence Interval

PPV: Positive Predictive Value

NPV: Negative Predictive Value

*: Significant Level at P value < 0.05

## Discussion

CAP is considered one of the most life-threatening infections in childhood, especially in developing countries where severe CAP is the leading cause of mortality resulting from infectious diseases [12].

Biomarkers could be useful in many areas in CAP: clarifying the etiology; assessment of the severity; prognosis; early admission for intensive care units; and proper selection of the management plan [13].

In this study, we elucidate the utility of biomarkers as RDW, Proadrenomedullin Pro ADM and Copeptin in association with PRESS for the prediction of poor outcomes of CAP in 99 children suffering from CAP (72 with uncomplicated CAP, and 27 with complicated CAP).

The mean values of RDW, Pro ADM, and Copeptin were significantly higher in the complicated group than in the uncomplicated group. Depending on further analysis of these biomarkers in the complicated CAP group, using simple logistic regression analysis, we found that Pro AMD was the most accurate biomarker, followed by RDW, length of hospital stay, and lastly by Copeptin for the prediction of complications of CAP in children. Also, variables that were significant in the simple logistic analyses were statistically re-analyzed via multiple stepwise logistic regression analyses that led to confirming the statistical significance of RDW and the length of hospital stay. To determine the prognostic power of these biomarkers for the complications of CAP, ROC curves were done, which revealed that RDW > 15.25% has sensitivity of 92.6% and specificity of 83.3% for the prediction of complications; and that RDW is superior over Pro ADM and Copeptin. So, we suggest that RDW could be used as a reliable prognostic biomarker for the complications of CAP in children. Our results are in harmony with Miranda (2017) [14] who concluded that there was a high level of association between elevated RDW and either mortality or the development of complications in children with CAP with specificity of 94.7% and sensitivity of 60% in the prediction of complications.

Similarly, Braun et al. [15], in their study of RDW in adult patients with CAP, found that high values of RDW on admission are significantly associated with mortality and severe morbidity in adult patients with CAP. This significant association between RDW and complicated CAP could be explained by the fact that RDW might be considered a significant biomarker for a high level of inflammatory activity generated in the state of CAP. Such an increase in the RDW levels is a result of cytokine release in response to inflammatory stress. These cytokines could block the activity of erythropoietin and suppress erythrocyte maturation that results in the production of ineffective red blood cells and elevated RDW [16].

In our study, Copeptin has an insignificant association with the included complications of CAP. Also, the prognostic power of Copeptin was less than RDW and Pro ADM. In contrast tour results, Abdel-Fattah et al.’s review [17] suggests that Copeptin could be a promising prognostic marker in CAP.

We found significant moderate correlations between RDW and PRESS. We use PRESS as it is easily applicable and useful for clinical assessment of the severity of the respiratory infection. Increasing the degree of the PRESS indicates an increase in the severity of pneumonia. So, the moderate association between RDW and PRESS means that, with the increase in the degree of severity of pneumonia, there is an increase in the values of RDW. Accordingly, RDW is a valuable indicator of the degree of severity of pneumonia. Also, the moderate correlation between Pro ADM and PRESS indicates that Pro ADM is similar to RDW in being an indicator of the degree of severity of pneumonia.

To predict mortality, a ROC curves model of the studied markers was done, in which RDW was found the most accurate prognostic biomarker at cutoff point > 17.4%, sensitivity of 81% and specificity of 89%. So, according to our results, RDW is an independent predictor of mortality.

Similarly, Miranda (2017) [14] found higher values of RDW in patients at admission associated with higher mortality. Also, in another study done by Karatas and Özyurt [18] on super-elderly patients with CAP, it was found that those basal RDW levels were correlated with mortality in super-elderly subjects with CAP and that RDW > 13.05% and age > 92 significantly increase mortality.

By analyzing the baseline characteristics of both the uncomplicated and complicated CAP groups, we found that a high percentage of patients (56.9%) in the uncomplicated group were males. In the complicated group, the percentage amounts to 70.4%.

Similar to our results, Muenchhoff and Goulder (2014) [19], Del Principe et al. (2013) [20] and Sánchez and González [21] found sex differences in pneumonia incidence. Muenchhoff and Goulder (2014) explained this male predominance either in complicated or simple CAP by suggesting that females have stronger humoral and cellular immune responses to infections or antigenic stimulation, which can be beneficial in protection and clearance of various pathogens. The mechanisms of this sex difference are multifactorial, including the endocrine and genetic effects on the immune system and physiology, in addition to sex-related differences in behavior [19].

This study has some limitations as it was conducted at one hospital. Also, RDW could be affected by iron, folate and vitamin B12, which were not included in this study. RBC transfusions are potential confounders on RDW values interpretations so we measure RDW before any RBC transfusions.

## Conclusion

Elevated RDW at admission in patients with CAP is a powerful predictor of complications and severity of pneumonia in children, and we suggest it be included in the admission criteria of PICU. Pro ADM is also a good predictor of admission to PICU. RDW helps in early identification of children with complicated CAP, and pediatricians can use RDW as a guide for decision making as regards admission to PICU and the early selection of appropriate management plans at EDs.

## Data Availability

The raw datasets of this study may be available from the corresponding author upon reasonable request.

## Abbreviations

RDW: Red Cell Distribution Width
pro-ADM: Pro-Adrenomedullin
CAP: Community-Acquired Pneumonia
PRESS: Pediatric Respiratory Severity Score
ED: Emergency Department
PICU: Pediatric Intensive Care Unit
